# Stigmatisation associated with COVID-19 in the general Colombian population

**DOI:** 10.1177/0020764020972445

**Published:** 2020-11-08

**Authors:** Carlos Arturo Cassiani-Miranda, Adalberto Campo-Arias, Andrés Felipe Tirado-Otálvaro, Luz Adriana Botero-Tobón, Luz Dary Upegui-Arango, María Soledad Rodríguez-Verdugo, María Elena Botero-Tobón, Yinneth Andrea Arismendy-López, William Alberto Robles-Fonnegra, Levinson Niño, Orlando Scoppetta

**Affiliations:** 1 Faculty of Health Sciences, Medicine Program, UDES Neuroscience Research Group Universidad de Santander, Bucaramanga, Colombia; 2 International Network for Stigma Reduction (RED_ESTIGMA); 3 Faculty of Health Sciences, Medicine Program, Health Psychology and Psychiatry Research Group, Universidad del Magdalena, Santa Marta, Colombia; 4 Faculty of Nursing, Care Research Group, Universidad Pontificia Bolivariana, Medellín, Colombia; 5 Institute of Medical Psychology and Medical Sociology, University Hospital of RWTH Aachen University, Aachen, Germany; 6 Mental Health and Addiction Department, Universidad de Sonora, Sonora, México; 7 Department of Preventive and Social Medicine, Pontificia Universidad Javeriana, Bogotá, Colombia; 8 Center for Innovation Culture and Society (CENICS); 9 Faculty of Psychology, GAEM Group (Research Methods Applied to Behavioral Sciences), Universidad Católica de Colombia, Bogotá, Colombia

**Keywords:** Social stigma, fear, Coronavirus infections (source MeSH)

## Abstract

**Background:**

As the COVID-19 pandemic progresses, the fear of infection increases and, with it, the stigma-discrimination, which makes it an additional problem of the epidemic. However, studies about stigma associated with coronavirus are scarce worldwide.

**Aims:**

To determine the association between stigmatisation and fear of COVID-19 in the general population of Colombia.

**Method:**

A cross-sectional study was carried out. A total of 1,687 adults between 18 and 76 years old (*M* = 36.3; *SD* = 12.5), 41.1% health workers, filled out an online questionnaire on Stigma-Discrimination and the COVID-5 Fear Scale, adapted by the research team.

**Results:**

The proportion of high fear of COVID-19 was 34.1%; When comparing the affirmative answers to the questionnaire on stigma-discrimination towards COVID-19, it was found that the difference was significantly higher in the general population compared to health workers in most of the questions evaluated, which indicates a high level of stigmatisation in that group. An association between high fear of COVID-19 and stigma was evidenced in 63.6% of the questions in the questionnaire.

**Conclusion:**

Stigma-discrimination towards COVID-19 is frequent in the Colombian population and is associated with high levels of fear towards said disease, mainly people who are not health workers.

## Introduction

The disease caused by SARS-CoV-2 (COVID-19) constitutes the most significant public health calamity in recent decades and imposes great medical, social, economic and political challenges (Wang & Flessa, 2020). Since the initial outbreak of pneumonia due to the new coronavirus, a rapid spread has occurred in the country and the world, with exponential growth in the number of infected people (Guan et al., 2020; Huang et al., 2020; Wang et al., 2020).

Around the world, the epidemiological behaviour of the pandemic was associated with significant levels of fear (Huang et al., 2020), and the appearance of stigmatisation related to the coronavirus (Lin, 2020), which has been expressed by avoidance behaviours, of rejection and, even, of psychological and physical violence against patients and health professionals (Bagcchi, 2020; Ng, 2020).

Regarding stigma, Ervin Goffman (2009) defines it as a characteristic or attribute that represents negative responses or unwanted effects for the person carrying it. Later, Link and Phelan (2001) defined it as a process that involves five components: (1) the labelling of human differences; (2) the dominant cultural beliefs that link people with undesirable characteristics and harmful stereotypes; (3) the location of people tagged in different categories (separation between them and us); (4) discrimination and a loss of status that stigmatised people experience; and (5) the exercise of the difference in economic, political and social power that characterises the stigmatisation process. The phenomenon of stigmatisation is conﬁgured when the prejudice adopts a negative social connotation (stereotype) (Katz, 1991) and that validates the hegemonic culture with behaviours that deny some right (discrimination) to the members of the group with the stigmatised characteristic (Klik et al., 2019).

Based on the labelling theory of Goffman (2009) and Scheff (1971), and the attribution theory of Weiner (1995), it is considered that stigma, prejudice, stereotype and discrimination overlap in a non-linear way (Cox et al., 2012; Major et al., 2002). This overlap has led them to be considered together as the stigma-discrimination complex (SDC), associated and inseparable concepts, which allows a more holistic look at this phenomenon (Campo-Arias & Herazo, 2015; Pescosolido & Martin, 2015). In this sense, the current pandemic generates another collateral phenomenon: The SDC associated with COVID-19 (SDC-CV-19) (Cassiani-Miranda & Campo-Arias, 2020).

While social distancing could be an effective way to reduce morbidity and mortality (Anderson et al., 2020), such distancing could inadvertently increase SDC towards affected populations (Bruns et al., 2020). As the COVID-19 pandemic progresses worldwide, the fear of infection increases and, with it, the stigma-discrimination of people, which makes it an additional problem of the epidemic (Devakumar et al., 2020). Thus, both health workers, people with active COVID-19 infection and their families, as well as those who have recovered from the disease face significant levels of stigma-discrimination (Singh & Subedi, 2020). It is possible that faced with a new disease, with severe symptoms and negative consequences such as death, fear of contagion and limited knowledge can generate a stigma-discrimination complete towards this disease (Lupia et al., 2020; Ren et al., 2020; Villa et al., 2020).

SDC-CV-19 is most likely the consequence of multiple ecological drivers (misinformation) and enablers (racism or poverty) (Logie & Turan, 2020; Phelan et al., 2008). Besides, disinformation through traditional media, and especially through social networks (Islam et al., 2020), may increase the risk of stigmatisation and undesirable behaviours (physical and psychological violence) (Sotgiu et al., 2020; Turner-Musa et al., 2020) towards the affected population.

In Latin America, the SDC-CV-19 has led to health workers as victims of threats, violence and intimidation (Taylor, 2020). SDC and xenophobia have historically accompanied epidemic outbreaks. For example, during the SARS outbreak of 2003, stigma-discrimination prevailed against Asian populations and, which increased the search for mental health care by these people (Person et al., 2004).

Regardless of the context, research has consistently shown that SDC is associated with adverse physical and mental health outcomes (Budenz et al., 2020; Budhwani & De, 2019; Ho et al., 2017; Karamouzian et al., 2018; Pachankis et al., 2018). Thus, such behaviours can undermine strategies to mitigate the disease and can lead to denial of medical care, lead to people not having the diagnostic test and not practising healthy behaviours, such as wearing face masks (Bruns et al., 2020; Turner-Musa et al., 2020).

The SDC-CV-19 has added burden to healthcare providers and administrators leading to more difficulties in tracking the contact of people infected with COVID-19, which could lead to an increase in cases and deaths from COVID-19 (Singh & Subedi, 2020; Sotgiu et al., 2020). Furthermore, the SDC-CV-19 can increase existing social inequalities by generating more unemployment and poverty and preventing various processes such as social integration (Shoib et al., 2020: Villa et al., 2020).

Therefore, addressing the SDC-CV-19 towards affected people and the highest risk groups has become a priority for medical and public health care providers (Bruns et al., 2020) and the need for the reduction of SDC-CV-19 as the most critical global challenges of the pandemic (‘Stop the Coronavirus Stigma Now’, 2020; Yoshioka & Maeda, 2020).

Consequently, understanding how the social determinants of health and SDC contribute to the incidence, treatment and mortality associated with COVID-19 can help the development of more effective interventions to mitigate the transmission of the disease (Sotgiu et al., 2020; Turner-Musa et al., 2020; Villa et al., 2020). Despite the urgent need to study SDC-CV-19 in-depth, primary studies specifically designed to evaluate the subject in the general population have been scarce in the world, especially in Latin America (Bagcchi, 2020). In Colombia, there is a study carried out on general practitioners, which found that 40% of doctors have felt discriminated and that 84% experience fear of COVID-19 (Monterrosa-Castro et al., 2020). However, there are no published studies in the general Latin American population on SDC-CV-19.

The present study aims to determine the association between experiences of stigmatisation associated with COVID-19 and fear of the coronavirus in the general population of Colombia.

## Methods

### Study design

An observational, analytical and cross-sectional study was carried out. A convenience sample was obtained from the general population of Colombia, and it included those over 18 years of age who voluntarily wanted to respond to an online survey on stigmatisation related to COVID-19.

### Measurements

#### Demographic characteristics

Variables such as age, sex, marital status, level of education and occupation were investigated through an electronic, anonymous and confidential survey designed by the researchers.

#### Questionnaire on COVID-19 Stigma-Discrimination

The questionnaire is made up of 11 items with dichotomous answers ‘yes’ or ‘no’, of these, seven were constructed from the scale of stigma towards tuberculosis (Upegui-Arango & Orozco, 2014, 2019), and the four additional items were designed by the authors of the present study, which represented more specific aspects of COVID-19 infection and its epidemiological dynamics. Since the homogeneity tests and the factorial analysis did not show satisfactory indices for the 11 items evaluated (Cronbach’s alpha of 0.51), the results of the questionnaire are presented with each question independently and not as a scale that measures a latent construct.

#### COVID-19 Fear Scale

The COVID-19 Fear Scale was adapted from the original 7-item scale developed by Ahorsu et al. (2020), after the adaptation and psychometric evaluation process carried out by the researchers of this study, it was conducted to obtain of a 5-item version (COVID-5 fear scale), which showed high internal consistency in this sample, with a Cronbach’s alpha of 0.75. The fear of COVID-5 scale has four response options, and the total scores are between 0 and 15. The distribution of the data defined high fear of COVID-19; scores equal to or higher than four, corresponding to the third quartile, were categorised as high fear related to Covid-19.

### Process

To collect the information, the researchers sent through emails and publications on their social networks (WhatsApp, Instagram and Facebook) an invitation to participate in the study to those interested, who had to fill out a self-administered online survey that it had an average completion duration of 7 minutes. Prior approval was obtained by the Research Ethics Committee of the Universidad del Magdalena.

### Statistical analysis

The description of the variables with frequencies and percentages was carried out for the qualitative data, and mean with standard deviation for the quantitative data. Measures of central tendency, dispersion and position were calculated according to the distribution of the COVID-19 fear scale scores. Subsequently, responses on the Stigma-Discrimination questionnaire related to COVID-19 and a high level of fear of COVID-19 were compared according to the occupation. Odds ratios with their 95% confidence intervals were calculated. The analysis was completed using the SPSS version 24 program.

## Results

A total of 1,687 adults between 18 and 76 years of age participated (*M* = 36.3, *SD* = 12.5). A total of 993 people from the general population and 694 workers in the health care. See more information in Table 1. Scores on the COVID-5 fear scale were observed between 0 and 15 (*M* = 2.96, *SD* = 2.63, Me = 2.0, IQR = 1.0–4.0), 34.1% scored for high COVID-19 fear.

**Table 1.** Demographical characteristics of sample (*N* = 1,687).

Variable	*n*	%
Age (years)		
18–29 (emerging adults)	567	33.6
30–44	710	42.1
45–64	379	22.5
65 o more	31	1.8
Gender		
Female	966	59.0
Male	691	41.0
Education		
High school or less	211	12.8
College or university	859	50.9
Postgraduate	617	36.3
Marital status		
Free union	281	16.7
Married	467	26.7
Separated or widowed	125	7.3
Single	814	48.3
Employee		
Yes	995	59.0
No	692	41.0
Kind of work		
General	993	58.9
Health	694	41.1
Fear COVID-19		
High (four or more on Fear-COVID-5)	575	34.1
Low	1,112	65.9

The frequencies of affirmative responses on the stigma-discrimination questionnaire and comparison, according to the occupation, are presented in Table 2. The difference was significantly more significant in the general population than health care workers in seven of the eleven questions. Likewise, it was observed that seven questions from the stigma-discrimination questionnaire (63.6%) were associated with a high COVID-19 fear. See the associations in Table 3.

**Table 2.** Affirmative response (%) in questionnaire stigma towards COVID-19 according to the occupation.

Item	General (*n* = 993)	Health (*n* = 694)	Total	OR (95% CI)
1. All foreigners are at higher risk of transmitting COVID-19	42.3	35.4	39.5	1.34 (1.09–1.63)
2. COVID-19 is a divine punishment	8.1	3.7	6.3	2.25 (1.43–3.54)
3. People should fear those who are sick with COVID-19	25.2	17.6	22.1	1.58 (1.24–2.01)
4. People sick with COVID-19 must be afraid to tell others that they have the disease	11.1	9.5	10.4	1.19 (0.86–1.64)
5. When I see news and stories about COVID-19 on television, press or social media, I get nervous or anxious	43.4	42.9	43.2	1.02 (0.84–1.24)
6. It is embarrassing to be sick with COVID-19	4.4	2.7	3.7	1.65 (0.95–2.85)
7. People get sick with COVID-19 because of irresponsible behaviour	56.1	48.1	52.8	1.38 (1.13–1.67)
8. People who work in health services and are in contact with COVID-19 patients should be isolated from society	12.4	5.8	9.7	2.31 (1.60–3.35)
9. People sick with COVID-19 should be rejected by society	0.4	0.4	0.4	0.93 (0.21–4.18)
10. People sick with COVID-19 can be neighbours of those who do not have this disease	13.3	7.3	10.8	1.93 (1.38–2.17)
11. I am afraid of being infected by the health personnel I meet in public transport, on the street or at my home	21.5	14.3	18.5	1.64 (1.26–2.13)

**Table 3.** Association between stigma-discrimination and high fear of COVID-19.

Item	OR (95% CI)
1. All foreigners are at higher risk of transmitting COVID-19	1.58 (1.29–1.94)
2. COVID-19 is a divine punishment	2.20 (1.48–3.27)
3. People should fear those who are sick with COVID-19	2.00 (1.58–2.53)
4. People sick with COVID-19 must be afraid to tell others that they have the disease	2.21 (1.62–3.03)
5. When I see news and stories about COVID-19 on television, press or social media, I get nervous or anxious	7.43 (5.92–9.33)
6. It is embarrassing to be sick with COVID-19	2.35 (1.42–3.89)
7. People get sick with COVID-19 because of irresponsible behaviour	1.19 (0.97–1.46)
8. People who work in health services and are in contact with COVID-19 patients should be isolated from society	1.18 (0.84–1.64)
9. People sick with COVID-19 should be rejected by society	1.45 (0.32–6.51)
10. People sick with COVID-19 can be neighbours of those who do not have this disease	1.13 (0.82–1.56)
11. I am afraid of being infected by the health personnel I meet in public transport, on the street or at my home	2.82 (2.19–3.63)

## Discussion

The results indicate a high level of stigmatisation in most of the situations evaluated. Also, an association between high fear of COVID-19 and stigma was evidenced in most of the questions in the questionnaire, especially in people with work occupations not related to the health care area and with a high level of COVID-19 fear.

The association between high fear and stigma related to the perception of the evaluated population, about the greater risk of transmission of COVID-19 by foreigners (OR = 1.58, 95% CI 1.29–1.94) is consistent with previous studies that have reported association of previous outbreaks of infectious diseases (e.g. influenza A (H1N1), bubonic plague, Asian flu, cholera, Ebola virus disease, Zika virus, HIV, tuberculosis, SARS and MERS) with levels of fear, stigma and discrimination against some populations (Fischer et al., 2019). For example, during the SARS outbreak in 2003, the National Center for Infectious Diseases of the Center for Disease Control and Prevention formed a multidisciplinary community group as part of its global response to the SARS emergency. This team monitored stigmatising ideas and behaviours in the general population and the media, particularly towards of Asian origin, finding in the respondents a disproportionate level of fear, stigmatisation and discrimination towards this population (Person et al., 2004).

Most of the stigmatisation experiences related to COVID-19 in the present study (level of stigma in the general population of 42.3%, and health care workers of 35.4% related to the perception in these population groups about the greater risk of transmission of COVID-19 by foreigners), are similar to the experience of stigmatisation processes that occurred during outbreaks of previous infections (Fischer et al., 2019). This situation suggests the need for monitoring and intervention to reduce the levels of stigma towards these populations, especially those with a high level of social vulnerability, such as the Venezuelan migrant population. In the context of the COVID-19 epidemic in Colombia and other countries in the southern cone of Latin America, Venezuelan people would be highly exposed to COVID-19 infection in many cases due to their homeless condition, and limited access to basic hygiene practices such as frequent hand washing, as well as a low nutritional level and even high levels of fear and stigma within these migrant groups that could limit their practices for the prevention of contagion, the transmission of the virus and the recognition of their rights as citizens (Fernández-Niño & Orozco, 2018). Situations such as those previously described reflect the existence of a series of pre-existing stigmas, which were amplified during the development of the COVID-19 pandemic, found new spaces to deploy or joined those created by this new situation (Turner-Musa et al., 2020).

On the other hand, health care workers linked to the care of patients diagnosed with COVID-19 have experienced and continue to experience SDC-CV-19, which has made them victims of experiences of discrimination such as the denial of entry to their homes, verbal abuse or the generation of rumours against them and social devaluation. Added to this, their family and friends are experiencing ‘secondary’ or ‘associative’ stigma. (Villa et al., 2020). This point was evidenced in the present study by associating experiences of stigmatisation towards health worker as ‘I feel a fear of being infected by the health personnel I meet in public transport, on the street or at home’ with fear of COVID-19 infection (OR = 2.82; 95% CI 2.19–3.63), which indicates a strong association between the high level of fear of the disease and stigmatising attitudes towards health workers, which must be taken into consideration in the framework of controlling the epidemic by COVID-19.

Most of the stigmatisation experiences described in this study were significantly associated with a high level of fear of COVID-19. These findings go in the same direction as a study conducted with psychiatrists from several countries from real-life experiences; they found that SDC-CV-19 was associated with similar factors (e.g. fear associated with infection or quarantine), beliefs (supra-natural or religious), shame and blame on oneself or others for contracting the disease (Villa et al., 2020) Additionally, the ‘infodemic’ (excessive circulation of misinformation) played as a driving factor and SDC-CV-19 facilitator (Ransing, Adiukwu et al., 2020). This aspect was represented in the present study as ‘When I see news and stories about COVID-19 on television, press or social networks, I get nervous or anxious’, and it was positively associated with fear of COVID-19 (OR = 7.43; 95% CI 5.92–9.33).

Due to the current reach of social networks and the mass media and immediate global communication through the Internet, during the COVID-19 pandemic, the stigmatisation phenomena promoted by these networks can reach even more significant proportions, even in populations with academic training in the health area. As could be observed in the present study, when evaluating the level of stigma between the general population and health care personnel, the percentage of stigma-stigmatisation expressed by both populations about this aspect was very similar, without a statistical significance in the probabilities of having a higher level of stigma according to the population group (level of stigma in the general population of 43.4%, and health care workers 42.9%, OR = 1.02; CI95% 0.84–1.24) therefore, The ‘infodemic’ becomes an essential factor associated with the stigma that must be studied in greater detail, in order to characterise its effects on various populations, including populations such as health care workers. Since they have a fundamental role in the control of diseases, such as COVID-19 (Islam et al., 2020).

Fear and its association with stigmatisation constitute a phenomenon that shows the high impact of the COVID-19 pandemic from a public health perspective (Dubey et al., 2020). Thus, previous studies have shown that fear associated with stigma and discrimination has negatively affected public health efforts with chronic conditions such as mental illness, HIV/AIDS, tuberculosis, leprosy, and epilepsy (Carey et al., 1997; Herek, 2002; MacLeod & Austin, 2003; Schulze & Angermeyer, 2003; WHO, 2003). For example, the stigmatisation associated with fear and the AIDS epidemic has negatively influenced voluntary testing, counselling and treatment of those infected with the disease (Chesney & Smith, 1999), revealing the adverse effects of the stigma/fear in the framework of control of several diseases. Being the case of the epidemic by COVID-19 a similar scenario, possibly with high sensitivity to the effects of these phenomena in crucial aspects of the control of the epidemic, which in light of the results of the present study are reflected, for example, in the association evidenced between aspects such as ‘People sick with COVID-19 should feel afraid of telling others that they have this disease’ and a higher level of fear of the disease (OR = 2.21; 95% CI 1.62–3.03). This specific association could lead to the concealment of the disease, limiting, for example, epidemic control activities such as the identification of contacts, and even, in those individuals with symptoms, but without a diagnosis, it could limit the search for medical attention (Ornell et al., 2020). Situations that, in addition to limiting control, could generate the rapid spread of the virus in the absence of a timely response (Harper et al., 2020). Therefore, interventions to reduce the SDC-CV-19 are increasingly necessary for the adequate control of the COVID-19 pandemic (Bagcchi, 2020).

The results of this study should be analysed in light of the following limitations. The cross-sectional nature of the study does not allow establishing a causality criterion between the reported associations, so subsequent studies such as cohorts should be carried out to establish a causal association between occupation, fear and the stigma associated with COVID-19. The fact that the sample is not probabilistic does not allow making inferences from the results to the general population of Colombia. Studies with probabilistic samples are recommended to establish the real prevalence of the phenomenon in Colombia.

Additionally, since there was no specific scale to measure stigma towards COVID-19, seven items from a pool of 25 items previously validated were adapted to measure stigma towards tuberculosis, and four items oriented to the context of the COVID-19 epidemic were added. These questions allowed evaluating some experiences of stigmatisation; however, it did not guarantee a reliable measure of the complex construct of stigma discrimination related to COVID-19. Therefore, it is recommended to design specific valid and reliable scales that more accurately measure the complex stigma discrimination towards COVID-19 as a construct, in the same way as fear of COVID-19 was evaluated with the specific fear scale to COVID-5. However, the findings presented here provide novel and crucial information on the dynamics of the SDC-CV-19 in a Latin American context. Although the measurement of experiences related to stigma was not carried out using a specific and valid scale concerning COVID-19, the statements evaluated allowed identifying critical aspects of the dynamics of stigma-discrimination in the population studied. This information allows not only to generate hypotheses of the possible associations between the phenomena of stigma and fear of COVID-19, but also to raise questions to consider in future research, such as the different mechanisms by which the infodemic exerts its effects on the levels of stigma towards COVID-19 in various populations, especially in the health care area.

Additionally, the results allowed the general exploration of specific situations in the current regional context, such as forced migration and the stigmatisation of these people (Keys et al., 2019) and the risk of transmitting COVID-19, a situation that could be a determining factor for the mitigation of the epidemic in places with high migrant traffic such as borders. If not addressed, the SDC-CV-19 can have severe consequences on the health outcomes of the population and the well-being of the community (Villa et al., 2020). Therefore, health professionals must understand the importance of protecting public health with strategies aimed at preventing fear, stigmatisation and discrimination associated with infectious diseases such as COVID-19 (Person et al., 2004).

Preventing SDC-CV-19 in a cultural context as complex as that of Latin America is a difficult task. Prevention depends on controlling or treating the coronavirus itself, increasing the level of knowledge about the disease, countering the tendencies of those who stigmatise others and supporting those who are stigmatised through emotional support and social policies (Rao et al., 2019). This complex task warrants an intersectoral and multilevel approach that could well be achieved through work in scientific networks (Eaton & Kalichman, 2020). The data collected during the evaluation of stigmatisation situations is the first input to guide the development of informed intervention strategies since it generates a characterisation of the magnitude of the phenomenon and associated variables in each specific cultural context (Stangl et al., 2019).

Further studies are needed on stigmatisation during the final phases of the pandemic and post-pandemic, including the analysis of perceptions and other qualitative categories that help to understand the phenomenon in a more holistic way, better understand the needs of communities and identify critical sources of information within populations to facilitate strategies with a participatory approach (Eaton & Kalichman, 2020). Likewise, establish the association between the SDC-CV-19, level of knowledge and perceptions, myths and rumours, as well as role of culture in stigma process, the implications in the process of seeking help, and other elements such as the impact of the media in generating stigmatisation. Besides, we believe that researchers working on the stigma associated with COVID-19 should develop and standardise stigma measures, which will allow them to compare outcomes and help to build more effective cross-cultural interventions (Ransing, Ramalho et al., 2020).

## Conclusion

It is concluded that SDC-CV-19 is frequent in the Colombian population and is associated with a high level of fear of COVID-19 and occurs in a higher proportion in people who are not health workers compared to the latter.

## References

[bibr1-0020764020972445] Ahorsu D. K. Lin C.-Y. Imani V. Saffari M. Griffiths M. D. Pakpour A. H. (2020). The fear of COVID-19 scale: Development and initial validation. International Journal of Mental Health and Addiction, 1–9 Advance online publication. 10.1007/s11469-020-00270-8 PMC7100496 32226353

[bibr2-0020764020972445] Anderson R. M. Heesterbeek H. Klinkenberg D. Hollingsworth T. D. (2020). How will country-based mitigation measures influence the course of the COVID-19 epidemic? The Lancet, 395(10228), 931–934. 10.1016/S0140-6736(20)30567-5 PMC7158572 32164834

[bibr3-0020764020972445] Bagcchi S. (2020). Stigma during the COVID-19 pandemic. The Lancet Infectious Diseases, 20(7), 782. 10.1016/S1473-3099(20)30498-9 32592670 PMC7314449

[bibr4-0020764020972445] Bruns D. P. Kraguljac N. V. Bruns T. R. (2020). COVID-19: Facts, cultural considerations, and risk of stigmatization. Journal of Transcultural Nursing, 31(4), 326–332. 10.1177/1043659620917724 32316872 PMC7324134

[bibr5-0020764020972445] Budenz A. Klassen A. Purtle J. Yom Tov E. Yudell M. Massey P. (2020). Mental illness and bipolar disorder on Twitter: Implications for stigma and social support. Journal of Mental Health, 29(2), 191–199. 10.1080/09638237.2019.1677878 31694433

[bibr6-0020764020972445] Budhwani H. De P. (2019). Perceived stigma in health care settings and the physical and mental health of people of color in the United States. Health Equity, 3(1), 73–80. 10.1089/heq.2018.0079 30915422 PMC6434589

[bibr7-0020764020972445] Campo-Arias A. Herazo E. (2015). El complejo estigma-discriminación asociado a trastorno mental como factor de riesgo de suicidio. Revista Colombiana de Psiquiatría, 44(4), 243–250. http://www.scielo.org.co/pdf/rcp/v44n4/v44n4a08.pdf 26578476 10.1016/j.rcp.2015.04.003

[bibr8-0020764020972445] Carey J. W. Oxtoby M. J. Nguyen L. P. Huynh V. Morgan M. Jeffery M. (1997). Tuberculosis beliefs among recent Vietnamese refugees in New York State. Public Health Reports, 112(1), 66–72. https://www.ncbi.nlm.nih.gov/pmc/articles/PMC1381842/9018292 PMC1381842

[bibr9-0020764020972445] Cassiani-Miranda C. A. Campo-Arias A. (2020). Stigma-discrimination: Significant collateral damage of COVID-19. Indian Journal of Psychiatry, 62, 610–611. 33678860 10.4103/psychiatry.IndianJPsychiatry_506_20 PMC7909044

[bibr10-0020764020972445] Chesney M. A. Smith A. W. (1999). Critical delays in HIV testing and care: The potential role of stigma. American Behavioral Scientist, 42(7), 1162–1174. 10.1177/00027649921954822

[bibr11-0020764020972445] Cox W. T. L. Abramson L. Y. Devine P. G. Hollon S. D. (2012). Stereotypes, prejudice, and depression: The integrated perspective. Perspectives on Psychological Science, 7(5), 427–449. 10.1177/1745691612455204 26168502

[bibr12-0020764020972445] Devakumar D. Shannon G. Bhopal S. S. Abubakar I. (2020). Racism and discrimination in COVID-19 responses. The Lancet, 395(10231), 1194. 10.1016/S0140-6736(20)30792-3 PMC7146645 32246915

[bibr13-0020764020972445] Dubey S. Biswas P. Ghosh R. Chatterjee S. Dubey M. J. Chatterjee S. Lahiri D. Lavie C. J. (2020). Psychosocial impact of COVID-19. Diabetes & Metabolic Syndrome: Clinical Research & Reviews, 14(5), 779–788. 10.1016/j.dsx.2020.05.035 PMC7255207 32526627

[bibr14-0020764020972445] Eaton L. A. Kalichman S. C. (2020). Social and behavioral health responses to COVID-19: Lessons learned from four decades of an HIV pandemic. Journal of Behavioral Medicine, 43(3), 341–345. 10.1007/s10865-020-00157-y 32333185 PMC7182505

[bibr15-0020764020972445] Fernández-Niño J. Orozco K. (2018). Migración venezolana en Colombia: Retos en Salud Pública. Revista de la Universidad Industrial de Santander, 50(1). Recuperado 5 de mayo de 2020 de, https://revistas.uis.edu.co/index.php/revistasaluduis/article/download/7992/8516?inline=1

[bibr16-0020764020972445] Fischer L. S. Mansergh G. Lynch J. Santibanez S. (2019). Addressing disease-related stigma during infectious disease outbreaks. Disaster Medicine and Public Health Preparedness, 13(5–6), 989–994. 10.1017/dmp.2018.157 31156079 PMC6889068

[bibr17-0020764020972445] Goffman E. (2009). Estigma: La identidad deteriorada. Amorrortu.

[bibr18-0020764020972445] Guan W. Ni Z. Hu Y. Liang W. Ou C. He J. Liu L. Shan H. Lei C. Hui D. S. C. Du B. Li L. Zeng G. Yuen K.-Y. Chen R. Tang C. Wang T. Chen P. Xiang J. , et al. (2020). Clinical characteristics of coronavirus disease 2019 in china. New England Journal of Medicine, 382(18), 1708–1720. 10.1056/NEJMoa2002032 PMC7092819 32109013

[bibr19-0020764020972445] Harper C. A. Satchell L. P. Fido D. Latzman R. D. (2020). Functional fear predicts public health compliance in the COVID-19 pandemic. International Journal of Mental Health and Addiction. Advance online publication. 10.1007/s11469-020-00281-5 PMC7185265 32346359

[bibr20-0020764020972445] Herek G. M. (2002). Thinking about aids and stigma: A psychologist’s perspective. The Journal of Law, Medicine & Ethics, 30(4), 594–607. 10.1111/j.1748-720X.2002.tb00428.x 12561266

[bibr21-0020764020972445] Ho C.-L. L. Pan W. Taylor L. D. (2017). Stigma of HIV testing on online HIV forums: Self-stigma and the unspoken. Journal of Psychosocial Nursing and Mental Health Services, 55(12), 34–43. 10.3928/02793695-20170905-01 28892555

[bibr22-0020764020972445] Huang C. Wang Y. Li X. Ren L. Zhao J. Hu Y. Zhang L. Fan G. Xu J. Gu X. Cheng Z. Yu T. Xia J. Wei Y. Wu W. Xie X. Yin W. Li H. Liu M. , et al. (2020). Clinical features of patients infected with 2019 novel coronavirus in Wuhan, China. The Lancet, 395(10223), 497–506. 10.1016/S0140-6736(20)30183-5 PMC7159299 31986264

[bibr23-0020764020972445] Islam M. S. Sarkar T. Khan S. H. Mostofa Kamal A.-H. Hasan S. M. M. Kabir A. Yeasmin D. Islam M. A. Amin Chowdhury K. I. Anwar K. S. Chughtai A. A. Seale H. (2020). COVID-19–related infodemic and its impact on public health: A global social media analysis. The American Journal of Tropical Medicine and Hygiene, 103, 1621–1629. 10.4269/ajtmh.20-0812 32783794 PMC7543839

[bibr24-0020764020972445] Karamouzian M. Knight R. Davis W. M. Gilbert M. Shoveller J. (2018). Stigma associated with sexually transmissible infection testing in an online testing environment: Examining the perspectives of youth in Vancouver, Canada. Sexual Health, 15(1), 46. 10.1071/SH17089 28859728

[bibr25-0020764020972445] Katz I. (1991). Gordon Allport’s ‘the nature of prejudice’. Political Psychology, 12(1), 125–157. 10.2307/3791349

[bibr26-0020764020972445] Keys H. M. Kaiser B. N. Foster J. W. Freeman M. C. Stephenson R. Lund A. J. Kohrt B. A. (2019). Cholera control and anti-Haitian stigma in the Dominican Republic: From migration policy to lived experience. Anthropology & Medicine, 26(2), 123–141. 10.1080/13648470.2017.1368829 29058456

[bibr27-0020764020972445] Klik K. A. Williams S. L. Reynolds K. J. (2019). Toward understanding mental illness stigma and help-seeking: A social identity perspective. Social Science & Medicine, 222, 35–43. 10.1016/j.socscimed.2018.12.001 30599434

[bibr28-0020764020972445] Lin C.-Y. (2020). Social reaction toward the 2019 novel coronavirus (COVID-19). Social Health and Behavior, 3(1), 1. 10.4103/SHB.SHB_11_20

[bibr29-0020764020972445] Link B. G. Phelan J. C. (2001). Conceptualizing stigma. Annual Review of Sociology, 27(1), 363–385. 10.1146/annurev.soc.27.1.363

[bibr30-0020764020972445] Logie C. H. Turan J. M. (2020). How do we balance tensions between COVID-19 public health responses and stigma mitigation? Learning from HIV research. AIDS and Behavior, 24(7), 2003–2006. 10.1007/s10461-020-02856-8 32266502 PMC7137404

[bibr31-0020764020972445] Lupia T. Scabini S. Mornese Pinna S. Di Perri G. De Rosa F. G. Corcione S. (2020). 2019 novel coronavirus (2019-nCoV) outbreak: A new challenge. Journal of Global Antimicrobial Resistance, 21, 22–27. 10.1016/j.jgar.2020.02.021 32156648 PMC7102618

[bibr32-0020764020972445] MacLeod J. S. Austin J. K. (2003). Stigma in the lives of adolescents with epilepsy: A review of the literature. Epilepsy & Behavior, 4(2), 112–117. 10.1016/S1525-5050(03)00007-6 12697134

[bibr33-0020764020972445] Major B. Quinton W. J. McCoy S. K. (2002). Antecedents and consequences of attributions to discrimination: Theoretical and empirical advances. Advances in Experimental Social Psychology, 34, 251–330. 10.1016/S0065-2601(02)80007-7

[bibr34-0020764020972445] Monterrosa-Castro A. Dávila-Ruiz R. Mejía-Mantilla A. Contreras-Saldarriaga J. Mercado-Lara M. Florez-Monterrosa C. (2020). Estrés laboral, ansiedad y miedo al COVID-19 en médicos generales colombianos. MedUNAB, 23(2), 195–213. 10.29375/01237047.3890

[bibr35-0020764020972445] Ng E. (2020). The pandemic of hate is giving COVID-19 a helping hand. The American Journal of Tropical Medicine and Hygiene, 102(6), 1158–1159. 10.4269/ajtmh.20-0285 32314701 PMC7253093

[bibr36-0020764020972445] Ornell F. Schuch J. B. Sordi A. O. Kessler F. H. P. (2020). ‘Pandemic fear’ and COVID-19: Mental health burden and strategies. Brazilian Journal of Psychiatry, 42(3), 232–235. 10.1590/1516-4446-2020-0008 32267343 PMC7236170

[bibr37-0020764020972445] Pachankis J. E. Hatzenbuehler M. L. Wang K. Burton C. L. Crawford F. W. Phelan J. C. Link B. G. (2018). The burden of stigma on health and well-being: A taxonomy of concealment, course, disruptiveness, aesthetics, origin, and peril across 93 stigmas. Personality and Social Psychology Bulletin, 44(4), 451–474. 10.1177/0146167217741313 29290150 PMC5837924

[bibr38-0020764020972445] Person B. Sy F. Holton K. Govert B. Liang A. , the NCID, SARS Community Outreach Team (listed in alphabetical order), Garza B. Gould D. Hickson M. McDonald M. Meijer C. Smith J. Veto L. Williams W. Zauderer L . (2004). Fear and stigma: The epidemic within the SARS outbreak. Emerging Infectious Diseases, 10(2), 358–363. 10.3201/eid1002.030750 PMC3322940 15030713

[bibr39-0020764020972445] Pescosolido B. A. Martin J. K. (2015). The stigma complex. Annual Review of Sociology, 41(1), 87–116. 10.1146/annurev-soc-071312-145702 PMC4737963 26855471

[bibr40-0020764020972445] Phelan J. C. Link B. G. Dovidio J. F. (2008). Stigma and prejudice: One animal or two? Social Science & Medicine, 67(3), 358–367. 10.1016/j.socscimed.2008.03.022 18524444 PMC4007574

[bibr41-0020764020972445] Ransing R. Adiukwu F. Pereira-Sanchez V. Ramalho R. Orsolini L. Teixeira A. L. S. Gonzalez-Diaz J. M. Pinto da Costa M. Soler-Vidal J. Bytyçi D. G. El Hayek S. Larnaout A. Shalbafan M. Syarif Z. Nofal M. Kundadak G. K. (2020). Mental health interventions during the COVID-19 pandemic: A conceptual framework by early career psychiatrists. Asian Journal of Psychiatry, 51, 102085. 10.1016/j.ajp.2020.102085 32413616 PMC7195073

[bibr42-0020764020972445] Ransing R. Ramalho R. Orsolini L. Adiukwu F. Gonzalez-Diaz J. M. Larnaout A. Pinto da Costa M. Grandinetti P. Bytyçi D. G. Shalbafan M. Patil I. Nofal M. Pereira-Sanchez V. Kilic O. (2020). Can COVID-19 related mental health issues be measured? Brain, Behavior, and Immunity, 88, 32–34. 10.1016/j.bbi.2020.05.049 PMC7248629 32470593

[bibr43-0020764020972445] Rao D. Elshafei A. Nguyen M. Hatzenbuehler M. L. Frey S. Go V. F. (2019). A systematic review of multilevel stigma interventions: State of the science and future directions. BMC Medicine, 17(1), 41. 10.1186/s12916-018-1244-y 30770756 PMC6377735

[bibr44-0020764020972445] Ren S.-Y. Gao R.-D. Chen Y.-L. (2020). Fear can be more harmful than the severe acute respiratory syndrome coronavirus 2 in controlling the coronavirus disease 2019 epidemic. World Journal of Clinical Cases, 8(4), 652–657. 10.12998/wjcc.v8.i4.652 32149049 PMC7052559

[bibr45-0020764020972445] Scheff T. J. (1971). Being mentally ill: A sociological theory. Transaction Publishers, p. 236.

[bibr46-0020764020972445] Schulze B. Angermeyer M. C. (2003). Subjective experiences of stigma. A focus group study of schizophrenic patients, their relatives and mental health professionals. Social Science & Medicine, 56(2), 299–312. 10.1016/S0277-9536(02)00028-X 12473315

[bibr47-0020764020972445] Shoib S. Nagendrappa S. Grigo O. Rehman S. Ransing R. (2020). Factors associated with COVID-19 outbreak-related suicides in India. Asian Journal of Psychiatry, 53, 102223. 10.1016/j.ajp.2020.102223 32574941

[bibr48-0020764020972445] Singh R. Subedi M. (2020). COVID-19 and stigma: Social discrimination towards frontline healthcare providers and COVID-19 recovered patients in Nepal. Asian Journal of Psychiatry, 53, 102222. 10.1016/j.ajp.2020.102222 32570096 PMC7293527

[bibr49-0020764020972445] Sotgiu G. Carta G. Suelzu L. Carta D. Migliori G. B. (2020, June 1). How to demystify COVID-19 and reduce social stigma. 10.5588/ijtld.20.0233 32553017

[bibr50-0020764020972445] Stangl A. L. Earnshaw V. A. Logie C. H. van Brakel W. C. Simbayi L. Barré I. Dovidio J. F. (2019). The Health Stigma and Discrimination Framework: A global, crosscutting framework to inform research, intervention development, and policy on health-related stigmas. BMC Medicine, 17(1), 31. 10.1186/s12916-019-1271-3 30764826 PMC6376797

[bibr51-0020764020972445] Stop the Coronavirus Stigma Now. (2020). Nature, 580(7802), 165–165. 10.1038/d41586-020-01009-0 32265571

[bibr52-0020764020972445] Taylor L. (2020). COVID-19 misinformation sparks threats and violence against doctors in Latin America. BMJ, m3088. 10.1136/bmj.m3088 32784200

[bibr53-0020764020972445] Turner-Musa J. Ajayi O. Kemp L. (2020). Examining social determinants of health, stigma, and COVID-19 disparities. Healthcare, 8(2), 168. 10.3390/healthcare8020168 PMC7349778 32545647

[bibr54-0020764020972445] Upegui-Arango L. D. Orozco L. C. (2014). Diseño de un instrumento para medir estigma hacia; la tuberculosis. Revista de la Universidad Industrial de Santander Salud, 46(1), 22–34.

[bibr55-0020764020972445] Upegui-Arango L. D. Orozco L. C. (2019). Estigma hacia la tuberculosis: Validación psicométrica de un instrumento para su medición. Anales de la Facultad de Medicina, 80(1), 12–20. 10.15381/anales.v80i1.15656

[bibr56-0020764020972445] Villa S. Jaramillo E. Mangioni D. Bandera A. Gori A. Raviglione M. C. (2020). Stigma at the time of the COVID-19 pandemic. Clinical Microbiology and Infection. Advance online publication. 10.1016/j.cmi.2020.08.001 PMC7411378 32777361

[bibr57-0020764020972445] Wang M. Flessa S. (2020). Modelling COVID-19 under uncertainty: What can we expect? The European Journal of Health Economics, 21(5), 665–668. 10.1007/s10198-020-01202-y 32494911 PMC7268590

[bibr58-0020764020972445] Wang W. Tang J. Wei F. (2020). Updated understanding of the outbreak of 2019 novel coronavirus (2019-nCoV) in Wuhan, China. Journal of Medical Virology, 92(4), 441–447. 10.1002/jmv.25689 31994742 PMC7167192

[bibr59-0020764020972445] Weiner B. (1995). Judgments of responsibility: A foundation for a theory of social conduct. Guilford Press, p. 326.

[bibr60-0020764020972445] World Health Organization. (2003). Leprosy: Urgent need to end stigma and isolation. Journal of Advanced Nursing, 42, 546–549. https://www.who.int/mediacentre/news/releases/2003/pr7/en/

[bibr61-0020764020972445] Yoshioka T. Maeda Y. (2020). COVID-19 stigma induced by local government and media reporting in Japan: It’s time to reconsider risk communication lessons from the Fukushima Daiichi nuclear disaster. Journal of Epidemiology, 30(8), 372–373. 10.2188/jea.JE20200247 32565498 PMC7348076

